# Nutrient Use Efficiency and Cucumber Productivity as a Function of the Nitrogen Fertilization Rate and the Wood Fiber Content in Growing Media

**DOI:** 10.3390/plants13202911

**Published:** 2024-10-17

**Authors:** Rita Čepulienė, Lina Marija Butkevičienė, Vaida Steponavičienė

**Affiliations:** Department of Agroecosystems and Soil Sciences, Vytautas Magnus University, K. Donelaičio Street 58, LT-44248 Kaunas, Lithuania; rita.cepuliene@vdu.lt (R.Č.); lina.butkeviciene@vdu.lt (L.M.B.)

**Keywords:** wood fiber, cucumber productivity, nutrient uptake, nitrogen fertilization, physicochemical properties of growing media

## Abstract

A peat substrate is made from peat from drained peatlands, which is a limited resource. A realistic estimate is that 50% of the world’s wetlands have been lost. Peat is used in horticulture, especially for the cultivation of vegetables in greenhouses. The consequences of peatland exploitation are an increase in the greenhouse effect and a decrease in carbon stocks. Wood fiber can be used as an alternative to peat. The chemical properties of growing media interact and change continuously due to the small volume of growing media, which is limited by the growing container. This study aims to gain new knowledge on the impact of nutrient changes in the microbial degradation of carbon compounds in wood fiber and mixtures with a peat substrate on the content and uptake of nutrients required by plants. The cucumber (*Cucumis sativus* L.) variety ‘Dirigent H’ developed in the Netherlands was cultivated in growing media of a peat substrate and wood fiber: (1) peat substrate (PS); (2) wood fiber (WF); (3) wood fiber and peat substrate 50/50 *v*/*v* (WF/PS 50/50); (4) wood fiber and peat substrate 25/75 *v*/*v* (WF/PS 25/75). The rates of fertilization were the following: (1) conventional fertilization (CF); (2) 13 g N per plant (N_13_); (3) 23 g N per plant (N_23_); (4) 30 g N per plant (N_30_). The experiment was carried out with three replications. As the amount of wood fiber increased, the humidity and pH of the growing media increased. The fertilization of the cucumbers with different quantities of nitrogen influenced the nutrient uptake. The plants grown in the 50/50 and 25/75 growing media had the best Cu uptake when fertilized with N_23_. When the plants grown in the wood fiber media and the 50/50 media were fertilized with N_13_, N_23,_ and N_30_, the Mn content in the growing media at the end of the growing season was significantly lower than the Mn content in the media with conventional fertilization. Thus, nitrogen improved the uptake of Mn by the plants grown not only in the wood fiber, but also in the combinations with a peat substrate. Growing plants in wood fiber and fertilizing them with N_13_ can result in the optimum uptake of micronutrients. The number and biomass of cucumber fruits per plant were influenced by the amount of wood fiber in the growing media and the application of nitrogen fertilizer. The highest number of fruits and biomass of fruits per plant obtained were significantly higher when the cucumbers were grown in WF/PS 50/50 growing media with additional N_13_ fertilization.

## 1. Introduction

The decline in arable land is putting pressure on agricultural producers. This is not only linked to changes in the ecosystem—soil degradation and climate change—but also to increasing urbanization. These changes are forcing farmers to look for alternatives to produce more from less land per unit area. These global changes have led to a significant increase in soilless crop production in recent years [1].

Soilless crop production can be 2–5 times more productive, with 10 times less water consumption compared to that of field production. This results in year-round production, a better taste, and a higher nutritional value [2]. Switching from an outdoor soil-based crop production system to a soilless system can improve water use efficiency, especially in closed-loop systems with a recirculating solution of water and nutrients that is collected in a closed drainage water tank for reuse [3,4]. Another reason is related to ecology and environmental protection; the intensive destruction of peatland areas that are important for the ecosystem and the use of artificial substrates cause waste accumulation problems and environmental pollution. Peat has been the dominant organic component of growing media in many parts of the world for the last 50 years due to its excellent physical properties, such as excellent air and water retention, low pH and salinity, and protection against pests and diseases [5]. Due to its ease of use and beneficial properties, peat has, over time, become the key component of growing media and still dominates the greenhouse sector. Worldwide, around 40 million m^3^ of peat is used annually in horticulture [6], with Germany (8.5 million m^3^) and the UK (2.5 million m^3^) being the main consumers of peat in horticulture [7]. Other resources, such as treated and untreated waste wood or its bark, can also be used as growing media or as the components of growing media, and renewable raw materials have a lot of potential for their use in agriculture for growing plants in closed systems without soil. The preliminary studies by Dutch researchers have shown that the performance of five peat–pine bark mixtures in the region is comparable to that of perlite at the vegetative and early fruiting stages of cucumbers [4,8,9]. Waste management strategies to reduce, reuse, and recycle waste should be further and more rigorously applied in soilless cropping systems [2,4,9]. Wood-based substrates, including pine bark and wood fiber, have good air permeability and high saturated hydraulic conductivity [10]. Wood-based substrates are more sustainable than peat and more readily available and cheaper than coir [8]. Wood fiber or sawdust is a by-product of the wood industry, produced from clean, broken wood. Wood substrates are typically produced by chopping and grinding a tree, as well as from the leaves, twigs, branches, and needles removed during forest thinning. Sawdust is a by-product of wood that remains after production operations and has been used as an ingredient in other growing media materials, especially where peat is scarce or expensive. Wood sawdust can be used as a component of growing media with peat [4,11]. Wood fiber is produced using mechanical defibrillation or, most commonly, water vapor by squeezing clean wood chips through a thermoplastic press. Wood fiber can be compressed for ease of transport [12]. Using wood fiber as a growing medium dates back to 2000 in Spain, France, and Germany [13]. New wood waste recycling plants have been established in the USA, Ireland, Germany, the UK, and Latvia [14]. Materials that are easy to produce, financially viable, environmentally friendly, and capable of providing quality growing media will become the future substitutes for rockwool and peat.

Climate change, soil degradation, and other ecological problems will drive the development of soilless cropping systems and the choice of growing media in the near future. Growing crops in soilless culture systems involves high energy consumption for heating in the cold season and artificial lighting, expensive greenhouse construction [15,16], fertilizer use [3], post-harvest transportation, and packaging [17]. On the other hand, SCSs (soilless culture systems) help to alleviate many of the problems associated with conventional in situ cultivation, such as soil-borne diseases and pests, and the precise control of water and fertilizer requirements [18,19]. The resulting high-quality production and higher yields in a shorter time justify the production costs in these systems [20].

Innovative, sustainable, and renewable materials, such as compost, bark, wood, coir, and other organic wastes, with low production costs and short transport routes may become the growing media of the future and dominate the market for growing media raw materials. In the future, waste and organic materials will be highly competitive materials, similar to wood and bark today [21]. As an alternative to synthetic and peat-based substrates, wood fiber from wood processing can be used, but further research is needed.

Nutrient availability is one of the main factors determining the suitability of organic substrates for plant growth [22], which may depend on their elemental composition and other factors affecting nutrient forms and dynamics, including the presence of dissolved organic compounds, the biological stability of the growing media, and the adsorption capacity [23]. The availability of nutrient elements, including magnesium and calcium, directly influences the rate of synthesis of certain metabolites in plants [24,25]. Wood fiber in growing media can influence the uptake of nutrients by a plant, as it has a nutrient storage function [26]. Nutrient cycling in wood fiber growing media is a complex and dynamic process influenced by various environmental factors and plant species, making it difficult to predict nutrient availability and uptake [27]. However, wood fiber is a suitable material for horticultural growing media due to its fibrous structure, hydrophilic nature, and low nutrient or growth-inhibiting content compared to those of other components such as bark [28].

Given the above, this study was carried out to identify a suitable soilless media made of wood fiber and peat, select optimum fertilization to compensate for nitrogen immobilization, and ensure optimum nutrient uptake and physicochemical properties for the growing media.

## 2. Materials and Methods

### 2.1. Site Description and Experiment Design

This experiment was conducted in Lithuania at Vytautas Magnus University Agriculture Academy, Joint Research Centre of Agriculture and Forestry, in a regulated-climate greenhouse and the Soil and Crop Ecology Laboratory of Experimental Station on 2021. The cucumbers were grown for 97 days. The cucumbers selected for the experiment with different growing media under controlled climate conditions were common short cucumbers (*Cucumis sativus* L.), variety ‘Dirigent H’, developed in the Netherlands. The cucumber seeds were sown in 28 × 38 cm plastic tray with 24 holes of 5.5 × 6 × 6 cm on 21 May 2021. The tray was filled with peat substrate for seed germination, consisting of a peat fraction of up to 7 mm. The substrate was enriched with 2.0 g L^−1^ fertilizer N_14_P_16_K_18_. The pH of the germination substrate was 6. The seedlings were grown from seeds in a climate-controlled chamber with lighting, RUMED1303. The temperature of the germination chamber was 25 °C, and the humidity was 80%. As the seedlings produced their second true leaf, they were transferred to the greenhouse and planted one plant at a time in 5 L plastic growing containers (D—23 cm (SBH)) filled with test growing media. Four growing media (four treatments of the experiment (factor A)) were studied: (1) 100% peat substrate (PS) (control substrate); (2) 100% wood fiber (WF); (3) 50/50% wood fiber/peat by volume (WF/PS 50/50); and (4) 25/75% wood fiber/peat by volume (WF/PS 25/75). The peat substrate was produced from a fraction of *Sphagnum* peat with a particle size <20 mm by the company Rekyva (Lithuania). Wood fiber was produced by Lithuanian company Nereta only from softwood highest-quality chips (residues of the lumber industry). Wood fiber is RAL-certified. Both the mixtures of wood fiber and peat were prepared just before the start of the experiment (company Rekyva, Lithuania). The wood fiber density was 0.16 g cm^−3^, the air dry moisture content was 41%, and the water absorption total was 2.1 g g^−1^. The chemical composition of the wood fiber and the peat substrate was analyzed in a laboratory of the Lithuanian Agricultural Advisory Service and in an agrochemical research laboratory of the Lithuanian Research Centre for Agriculture and Forestry. The characteristics of the materials used for the cucumber-growing media are presented in Table 1.

**Figure 1:**
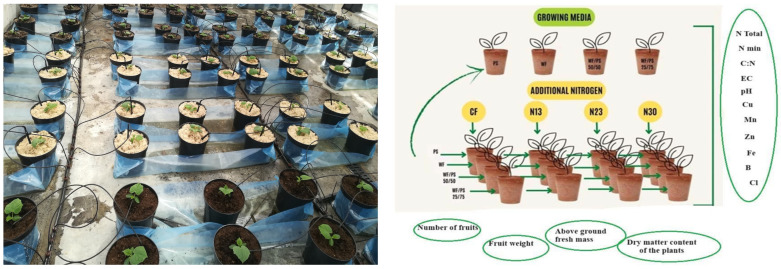
Experiment after transplanting cucumbers into growing containers and experimental design.

The growing containers were arranged in rows on either side of the irrigation pipe. Each row contained 12 growing containers spaced 30 cm apart. The whole experiment consisted of 16 rows, i.e., 192 growing containers. One irrigation capillary (drip sprinkler) was introduced into each growing container (Figure 1).

Each treatment consisted of 48 growing containers, with one cucumber plant each. Each treatment of the growing media (substrate) was divided into four groups with four fertilization backgrounds (factor B): (1) conventional fertilization (control); (2) N_13_ as an additional fertilization—13 g N per plant; (3) N_23_ as an additional fertilization—23 g N per plant; and (4) N_30_ as an additional fertilization—30 g N per plant.

The experiment was carried out with three replications. The growing containers were arranged in a completely randomized design.

The average daily temperature in the greenhouse during the experiment was 25 °C, the humidity was 65%, and the daily light integral was 21.8 mol·m^−2^ d^−1^. The cucumbers were watered every two hours. Water was supplied to each plant using a capillary irrigation system. The growing containers were protected with polyethylene film to prevent excess water from draining into the drainage system. The plants were grown as a single stem throughout the study. The cucumbers grew upwards using polypropylene ropes.

The experiment was carried out for 80 days after transplanting the seedlings into the greenhouse.

### 2.2. Plant Fertilization

**Figure 2:**
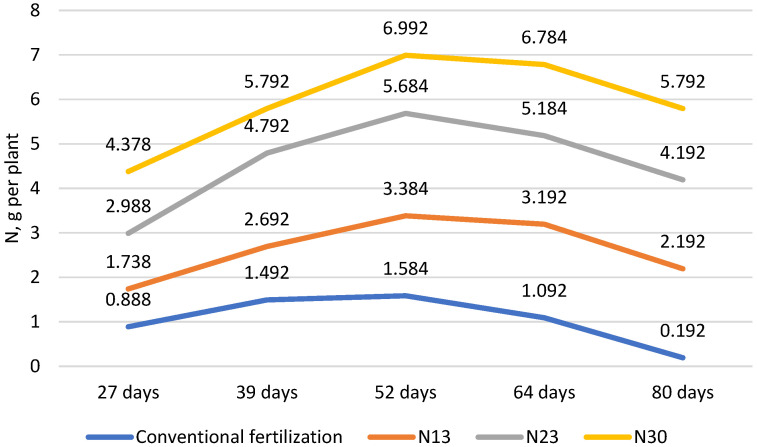
Nitrogen fertilization during cucumber growing season (days after transplanting). N13, N23, and N30—additional nitrogen fertilization.

In the experiment, conventional fertilization was carried out with the mineral fertilizer YaraMila^®^ COMPLEX NPK 12-11-18 with microelements. This complex fertilizer contains all the essential nutrients needed for the plants. The chemical composition of the fertilizer is as follows: nitrogen (N): 12%; phosphorus (P_2_O_5_/P): 11/4.8%; potassium (K_2_O/K): 18/14.9%; magnesium (MgO/Mg): 2.7/1.8%; sulfur (SO_3_^−^/S): 20/8%; boron (B): 0.015%; iron (Fe): 0.20%; manganese (Mn): 0.02%; and zinc (Zn): 0.02%. A total of 1.60 kg of complex fertilizer was dissolved in 1 m^3^ of water. Until they flowered, the cucumbers were fertilized once a week with 500 mL of the nutrient solution per plant. The cucumbers were fertilized twice a week with this fertilizer during the later stages of growth until the end of the growing season. For additional nitrogen fertilization, water-soluble calcium nitrate (Ca(NO_3_)_2_) was used. The chemical composition of the fertilizer was as follows: total nitrogen (N)—15.5% ((nitrate nitrogen (N–NO_3_)—14.4%) and ammonia nitrogen (N–NH_4_)—1.1%); and calcium oxide (CaO)—26.3% (calcium—18.8%). Calcium nitrate was applied once a week (11 times every 7 days) to the plants during the growing season to provide them with the additional nitrogen required in the experiment (Figure 2).

### 2.3. Measurement Properties of Growing Media

The agrochemical parameters of the growing media were determined at the end of the experiment. Samples of the growing media were taken from the two growing containers of each treatment after cutting the aboveground part of the plant for agrochemical analyses.

Organic carbon and nitrogen in the growing media were determined according to standard methods. Organic carbon (C) was determined using the Tyurin spectrophotometric method [24]. The sample was heated with a solution of potassium bichromate in sulfuric acid (+160 °C). The final measurement of C at 590 nm was carried out on a Cary 60 spectrophotometer (Varian, Palo Alto, CA, USA) using glucose standard solutions. The total nitrogen (N_total_) content of the soil was determined by the Kjeldahl method (the Kjeldahl apparatus Vapodest), with a spectrophotometric measurement at 655 nm. The mineral nitrogen (N_min_) content was determined spectrometrically using sulfosalicylic acid [29].

Copper (Cu) and zinc (Zn) contents were determined according to EN 13650:2003 and EN 8288:2002. Manganese (Mn) and total iron (Fe) were determined according to EN 13650:2003, AOAC 974.27. Boron (B) and chlorine (Cl) were determined using LST EN 13650:2003, LST EN ISO 11885:2009. Chemical analysis of nutrients was carried out at the Agrochemical Research Laboratory of the Lithuanian Research Centre for Agriculture and Forestry. All nutrients were determined in three replications.

The electrical conductivity (EC), density (D), and moisture content (M) of the growing media were measured with a Delta-T Device HH2 Moisture Meter (Cambridge, UK) at a depth of 10 cm in each growing container six times per growing season. The acidity (pH) of the growing media was determined by potentiometric determination in a 1 N KCl extract according to ISO 10390:2005.

The variation (%) in micronutrient content in the growing media during the growing season was calculated by summing the nutrient content of the growing media (dry matter) at the start of the experiment with the nutrient content of the fertilizer. This amount is equivalent to 100%. The micronutrient content (%) of the growing media (dry matter) at the end of the experiment, minus 100, was taken as nutrient uptake.

### 2.4. Plant Growth and Yield Measurements

Cucumber productivity was determined by picking, weighing, and counting the marketable fruit (17–20 cm) from each cucumber treatment separately. The cucumber fruits were harvested twice a week. Total cucumber productivity during the growing season was determined at the end of the experiment (80 days after transplanting) by converting the results obtained into productivity in kg per plant.

At the end of each stage of the study, the plants were cut from the two growing containers of each treatment, and the green biomass of the aboveground part was determined. The plants with all their morphological parts were weighed and crushed. The dry biomass of the aboveground part of the plant was determined by drying at 105 °C in a drying oven Memmert (Schwabach, Germany) to a constant biomass.

### 2.5. Statistical Analysis

All data were statistically analyzed using the computer program package STATISTICA10 using the method of two-way (growing media × fertilization rate) analysis of variance (ANOVA). Statistically significant differences in the data were determined by Fisher’s and the least significant difference (LSD) tests at the probability levels of 95% (*p* < 0.05) and 99% (*p* < 0.01). Principal component analysis (PCA) was performed using principal component and classification methods. PCA is a widely used statistical technique that simplifies complex multidimensional data into a smaller set of interpretable variables called principal components.

## 3. Results

### 3.1. Cucumber Productivity and Physicochemical Properties of Growing Media

The moisture content of the cucumber growing media depended on the amount of wood fiber and additional N, but significant differences were found in the non-factor-related interaction (growing media × fertilization rate) (Table 2). Increasing the amount of wood fiber in the media increased the moisture content from 9.8 to 35.0% compared to the moisture content of the peat substrate. Additional N fertilization significantly reduced the moisture content of the cucumber growing media by up to 37.6% compared to that of the growing media without additional fertilization. The density of the growing media was influenced only by its composition, decreasing with an increasing wood fiber content. Electrical conductivity (EC) depended on the interaction of the factors, on the proportion of wood fiber in the growing media, and on the addition of N fertilizer. The highest EC value was found in the growing media mixture WF/PS 25/75. The index decreased with an increasing fiber content in the media. The EC value in the wood fiber media was higher than that in the peat substrate, but lower compared to the EC value in the WF/PS 50/50 media. The additional fertilization with N_30_ increased the EC value by a factor from 1.1 to 3.3 compared to the EC value in the media without additional fertilization. A higher proportion of the wood fiber in the growing media had an alkalizing effect by increasing the pH, while the additional fertilizer N_13–30_ significantly decreased the pH of the media.

The dry biomass and green biomass of the cucumber plants were significantly influenced by the interaction of factors. The higher proportion of wood fiber in the media (WF/PS 50/50 and WF) and the additional fertilization with higher rates of N_23,30_ significantly reduced the dry biomass content of the aboveground biomass of the plants. The aboveground green biomass was significantly higher only when the cucumbers were grown on the peat substrate without additional N fertilization. Increasing the proportion of wood fiber and N fertilization in the growing media resulted in a significant decrease in the average aboveground green biomass of the plants. The amount of fruit and biomass of cucumber fruits on the plant were influenced by the amount of wood fiber in the growing media and additional N fertilization. The highest number of fruits was produced on the plant when they were grown in a 50/50 mixture of peat substrate and wood fiber WF/PS, with additional fertilization with the lowest rate of nitrogen N_13_. The cucumbers were also significantly higher in fruit biomass when grown in a WF/PS 50/50 mixture without additional fertilizer and with the lowest rate of additional N_13_.

**Figure 3:**
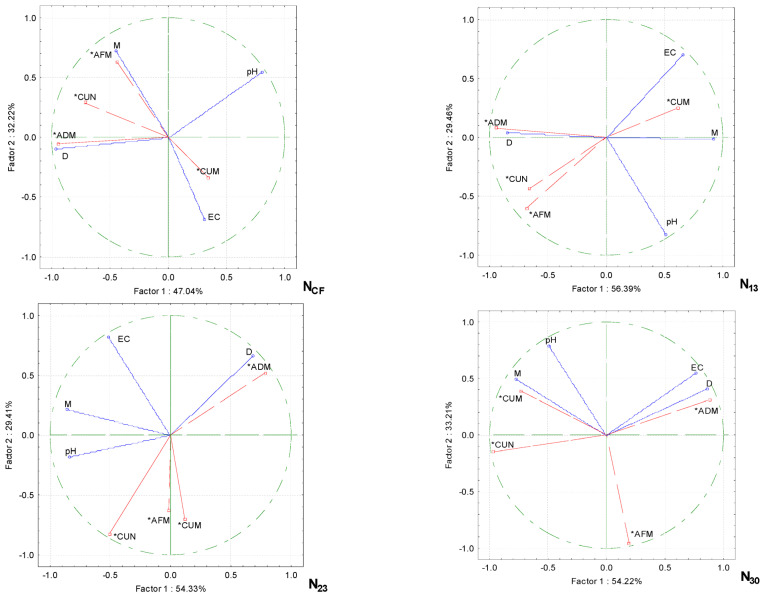
PCA analysis shows the relationship between the physicochemical properties of the growing media with wood fiber and the productivity parameters for the cucumbers grown under different nitrogen fertilization rates. Note: the density of the growing media (D), the electrical conductivity of the growing media (EC), the moisture of the growing media (M), the aboveground dry biomass content of the plants (ADM), the aboveground fresh biomass content of the plants (AFM), the number of cucumbers per plant (CUN), the cucumber biomass per plant (CUM), conventional fertilization (CF), the fertilization rates (N_13_, N_23_ and N_30_), and active variables (*).

Principle component analysis (PCA) showed that the physicochemical properties of the growing media were altered by increasing the proportion of wood fiber and the quantity of nitrogen fertilizer and that the productivity of cucumbers depended on them (Figure 3). Only the pH of the growing media had no significant effect on the cucumbers’ performance, irrespective of the rate of nitrogen fertilization. The other physicochemical properties of the growing media were correlated in distinct groups. When the cucumbers were grown without supplementary fertilization (CF), the green aboveground biomass and the fruit content of the plants correlated strongly with the moisture content of the growing media, while the dry biomass content of the aboveground biomass was influenced by the density of the media. There was a strong correlation between the EC of the growing media and the biomass of fruit on the plants, but a negative correlation was found between this and the moisture of the growing media.

With additional N_13_ fertilization, a strong correlation was obtained in the group between the EC of the growing media and the biomass of the fruit on the plants, with a weaker correlation with the moisture content of the media. The other strongly correlated group was the plant aboveground dry biomass and the density of the growing media. The cucumber fruit content and plant aboveground green biomass were negatively correlated with the EC of the growing media.

When the nitrogen fertilizer rate was increased to N_23_, only the dry biomass content of the aboveground plant correlated with the density of the growing media. The other cucumber performance parameters were not affected by the physical characteristics. When the cucumbers were fertilized at the maximum rate of N_30_, the largest group of correlated parameters was the aboveground dry biomass of the plant with the electrical conductivity and density of the growing media. In another group, there was a strong correlation between the cucumber fruit biomass and the moisture content of the growing media.

### 3.2. Micronutrient Content, Nitrogen Content, and C:N at the End of the Growing Season in the Growing Media

This study found that the highest uptake of micronutrients during the growing season was observed in the plants grown in the wood fiber (Table 3). The plants grown in this medium had a 37 percentage point higher copper uptake than the plants grown in the peat substrate with conventional fertilization. The plants grown in wood fiber showed the best uptake of zinc and manganese when fertilized with the highest N fertilization rate of N_30_, which was 13 and 28 percentage points, respectively, compared to that of conventional fertilization in wood fiber. Only the iron uptake of the plants grown in wood fiber decreased with increasing nitrogen levels. The plants fertilized with N_30_ nitrogen absorbed 15 percentage less iron than the plants grown in the wood fiber with conventional fertilization, but 6 percentage more than the plants grown on the peat substrate with conventional fertilization.

A higher nitrogen content N_30_ interfered with the uptake of copper and zinc by the plants grown on the peat substrate and on the WF/PS 50/5 and WF/PS 25/75 wood fiber media. The maximum N_30_ fertilization on the peat substrate reduced the zinc uptake by 39 percentage points, by 7 percentage points on the WF/PS 50/50 growing media, and by 21 percentage points on the WF/PS 25/75 growing media compared with the zinc uptake by the plants given the conventional fertilization. Thus, the plants grown in these growing media showed the best uptake of zinc under conventional fertilization. The plants grown in WF/PS 50/50 and WF/PS 25/75 had the best uptake of copper when fertilized with N_23_. Copper uptake increased by 2 and 16 percentage points, respectively, compared to that under conventional fertilization.

Manganese and iron uptake by the plants was better when fertilized with increased nitrogen. The plants grown on the peat substrate showed the best uptake of these trace elements when fertilized with N_13_ nitrogen, with increases of 19 and 15 percentage points, respectively, compared to that of conventional fertilization. The plants grown on the WF/PS 50/50 media showed the best uptake of manganese and iron when fertilized with N_23_ nitrogen. The plants grown on WF/PS 25/75 had the best uptake of manganese when fertilized with N_13_ nitrogen and iron when fertilized with N_23_ nitrogen. The cucumbers grown on the WF media showed a better uptake of Fe and Mn than the cucumbers grown on the other growing media studied. The cucumbers fertilized with conventional fertilizer (CF) in WF were 32 percentage points more efficient in Fe uptake than those in PS.

The trends of the boron and chlorine contents in the growing media were similar. The smallest changes in these elements during the growing season were observed in the media where the plants were fertilized with conventional fertilization. When the plants were fertilized with N_13_ nitrogen, the boron content of the growing media increased from 19 percentage points in the WF/PS 25/75 growing media to 45 percentage points in the wood fiber. Additionally the chlorine content increased from 23 percentage points in the WF/PS 50/50 growing media to 73 percentage points in the WF/PS 25/75 growing media compared with the levels of these elements in the media where the plants were treated with conventional fertilization. As the nitrogen supply to the plants was further increased up to the maximum rate of N_30_, the levels of these chemical elements increased in all the growing media studied, and at the end of the cucumber growing season, the levels of boron increased from 30 percentage points in WF to 80 percentage points in WF/PS 50/50. Additionally the level of chlorine increased from 106 percentage points in WF/PS 25/75 to 191 percentage points in PS compared with the level of the growing media where the plants were treated with conventional fertilization.

At the end of the cucumber growing season, the micronutrient content of the growing media was determined to assess how the plant nutrient uptake depended on the fertilization with different nitrogen rates and the amount of wood fiber in the growing media. The comparison of the copper content of all the media at all the fertilizer rates showed that at the end of the growing season, the WF media contained significantly less copper, by as much as 99%, and the WF/PS 25/75 media contained significantly more copper than the PS media under conventional fertilization (CF).

This study showed that the highest uptake of micronutrients during the growing season was observed for plants grown on WF. The plants grown on this medium had a 37-percentage-point-higher copper uptake than the plants grown on PS. The plants grown in the wood fiber had the best uptake of zinc and manganese when fertilized with the highest nitrogen rate N_30_, which was 13 and 28 percentage points, respectively, compared to that of the conventional fertilizer WF. Only the uptake of iron by WF plants decreased with increasing nitrogen levels. The plants fertilized with N_30_ absorbed 15 percentage less of it than those subjected to WF and conventional fertilization, but 6 percentage points more than those subjected to PS and conventional fertilization (CF). The boron content was more influenced by the composition of the media than by nitrogen fertilization. Boron uptake by the plants was better from PS and WF without additional nitrogen fertilizer. The higher nitrogen rate made the uptake of boron from the growing media more difficult.

At the end of the cucumber growing season, the highest levels of total and mineral nitrogen were found in the WF/PS 25/75 growing media, but the carbon/nitrogen ratio was found in the WF media (Table 4). PS had the lowest Fe content compared to those of the WF growing medium and the mixed medium, with an average of 1.9 times more of the content of this trace element. The total and mineral nitrogen contents of the growing media increased with increasing nitrogen fertilization. The C:N ratio was more dependent on the proportion of wood fiber in the growing media and decreased with an increasing nitrogen fertilizer rate up to N_30_.

**Figure 4:**
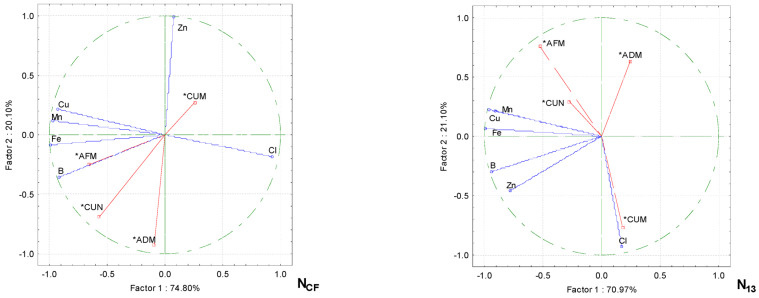

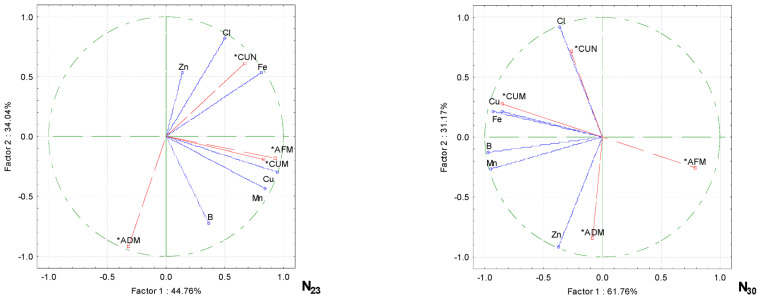
PCA analysis shows the relationship between the micronutrients on the growing media with wood fiber and the productivity parameters for the cucumbers under different nitrogen fertilization rates. Note: the density of the growing media (D), the electrical conductivity of the growing media (EC), the moisture of the growing media (M), the aboveground dry biomass content of the plants (ADM), the aboveground fresh biomass content of the plants (AFM), the number of cucumber fruits per plant (CUN), the number of cucumber fruits per plant (CUM), conventional fertilization (CF), the fertilization rates (N_13_, N_23_ and N_30_), and active variables (*).

Principle component analysis showed that increasing the proportions of wood fiber and nitrogen fertilizer in the growing media resulted in a change in the trace element content of the media, which, in turn, influenced the cucumbers’ performance. These parameters were correlated in separate groups (Figure 4). The green biomass of the cucumber plants in the substrate without additional fertilization (NCF) was strongly dependent in the group on B and Fe, and to a lesser extent on Mn and Cu. The cucumber fruit sets in pcs m^−2^ were more dependent on B than on the Fe content in the growing media and significantly less on Mn and Cu. The cucumber fruit biomass per plant was strongly correlated with Zn, but there was a negative correlation between this trace element and the plant dry biomass.

In the growing media, increasing the nitrogen rate (N_13_), the number of cucumber fruit pieces m^−2^, and the green biomass of the plants in kg m^−2^ correlated strongly with Mn and Cu and less strongly with the Fe content of the substrate. The number of cucumber fruits per plant (CUM) was strongly correlated with the Cl content of the growing media.

When the nitrogen fertilizer level was increased to N_23_, a correlation was observed in two groups. The cucumber fruit biomass in units m^−2^ was strongly correlated with Zn, Cl, and Fe, while in the other group, the cucumber plant green biomass and the fruit biomass were strongly correlated with Cu and Mn, with a weaker correlation with B. A negative correlation was found between the plant dry biomass and Zn.

When the fertilizer rate was increased to a maximum of N_30_, there was a very strong correlation between the fruit content and the Cl content, and the fruit biomass was strongly dependent on the Cu and Fe contents of the growing media. The dry biomass content of the aboveground part of cucumber plants at N_30_ was already positively correlated with the Zn content of the growing media. A negative correlation was found between the green aboveground biomass of the plants and the Cu and Fe contents of the media.

**Figure 5:**
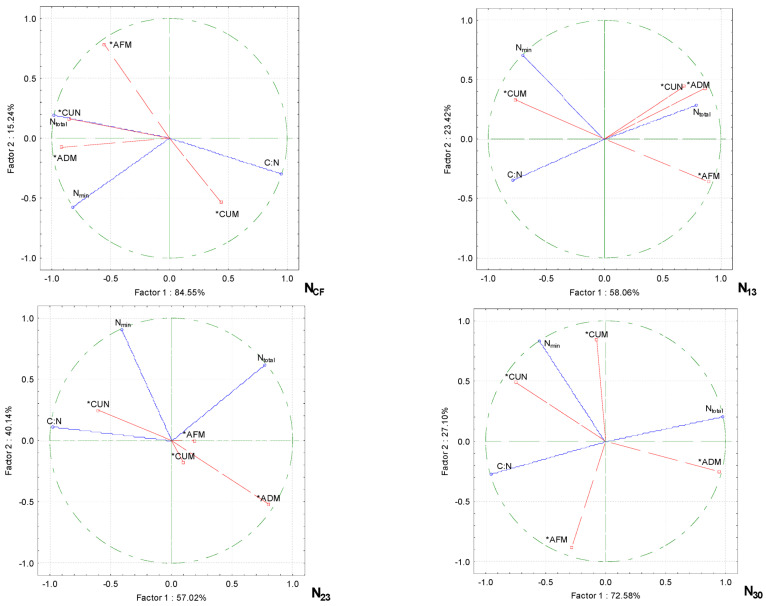
PCA analysis shows the relationship between the N_total_, N_min_, and C:N in the growing media with wood fiber on the productivity parameters for the cucumbers with different nitrogen fertilization rates. The density of the growing media (D), the electrical conductivity of the growing media (EC), the moisture of the growing media (M), the aboveground dry biomass content of the plants (ADM), the aboveground fresh biomass content of the plants (AFM), the number of cucumber fruits per plant (CUN), the number of cucumber fruits per plant (CUM), conventional fertilization (CF), the fertilization rates (N_13_, N_23_ and N_30_), and active variables (*).

Principle component analysis showed that increasing the proportion of wood fiber and nitrogen fertilizer in the growing media resulted in changes in the total and mineral nitrogen contents and the carbon/nitrogen ratio in the growing media and that these changes were related to the cucumber performance parameters (Figure 5). The fruit biomass was significantly influenced by the C:N ratio, while the other performance parameters were not affected by higher nitrogen rates and C:N. In the substrate without supplementary nitrogen (N_CF_), the number of cucumber fruits in pcs m^−2^ was strongly influenced by N_total_, while the dry biomass content of the aboveground part of the plants correlated not only with N_total_, but also with N_min_. When N fertilization was increased with N_13_, the cucumber fruit biomass depended on the N_min_ content, while the fruit content and the aboveground dry biomass correlated strongly with N_total_ in the growing media. The fruit content of the cucumbers on the N_min_ growing media was significantly influenced by the addition of more intensive N fertilization with N_23_. This was also strongly correlated with the C:N ratio. The cucumber green aboveground biomass was strongly correlated with the N_total_ content. A negative correlation was found between the biomass of fruit on the plant and the N_min_ in the substrate.

This research shows that the composition of the growing medium and the amount of fertilizer need to be carefully balanced to ensure optimum growing conditions for cucumbers. Combinations of wood fiber and peat with moderate rates of nitrogen can be effective for cucumber cultivation, providing better access to moisture and nutrients, while too much nitrogen can interfere with the uptake of nutrients and lead to problems of balanced plant nutrition.

## 4. Discussion

Plants grown under climate-controlled conditions take in more nutrients from the growing media than those grown outdoors. This high nutrient demand is due to the shallow penetration of the root system (limited by the volume of the growing container), the weak suction power of the roots, and the large difference between the biomass of the aboveground part of the plant and the root system. To provide the plants grown with wood fiber under controlled climatic conditions in different media with the required micronutrient content, the micronutrient content of the media at the beginning and the end of the study was determined. Wood fiber contains a significant amount of nutrients, and the concentration of these nutrients in wood varies between tree species and depends on the nutrient supply of the soil [29]. Fe use efficiency can contribute to improving plants’ physiology by playing the key role as a component of the catalytic centers of various bio-protein redox enzymes, including cytochromes, peroxidases, and catalases. This trace element is vital for nitrogen assimilation and photosynthesis [30].

Wood fiber, wood shavings, and sawdust are wastes from the wood industry. All these products have good water and air retention. Wood fiber can be used in crop production as a stand-alone substrate or in combination with other components such as peat [15,31]. Typically, wood fiber-enriched substrates are used for growing ornamental plants, shrubs, or seedlings in pots [32,33], but are also used in vegetable production [34]. The sorption capacity depends on many factors: the amount of wood fiber in the substrate, the physical and chemical properties of the fiber, and its type and origin [35,36]. In this study, the moisture absorption of the wood fiber/peat blend WF/PS 50/50 was significantly higher compared to that of PS and WF/PS 25/75 (Table 2). Fertilization with higher nitrogen rates of N_23_ and N_30_ reduced the moisture absorption of the growing media, but there was no interaction between media composition and fertilization. Principle component analysis showed that by increasing the proportion of fiber in the growing media, the green biomass of the cucumber plants correlated very strongly with the moisture content of the growing media without additional fertilization with NCF (Figure 3). The biomass of cucumber fruits on the plant correlated strongly with the additional fertilization at the highest rate of N_30_ [37], pointing out that the addition of 40% and 50% wood fiber to peat reduces the water content by 16% *vv*^−1^ and increases the air content of the substrate by the same amount. This indicates that it is more difficult to overwater, but also requires the more accurate monitoring of the water content and the state of the nutrients. Wood fiber media need to be watered more frequently than peat. *Pelargonium* grown in wood fiber was also found to flower earlier than the ones grown in other media or mixtures with wood fiber [11,14]. In our experiment, the cucumbers were irrigated equally in all the media. However, the moisture content was significantly highest in WF, but was reduced by additional fertilization with N_23_ and N_30_. Wood fiber has a high level of total porosity and, in most cases, a very high number of air-filled pores and a relatively low level of readily available water. It was found also to have a higher air diffusion rate compared to that of peat [11,38]. Therefore, wood fiber is used to optimize the physical properties of other components in growing media, e.g., by reducing the bulk density, increasing the air capacity, and improving the re-wetting capacity [16,39]. The density of the growing media was highly dependent on their composition. The density of the WF media and the WF/PS 50/50 mixture was significantly lower compared to that of PS. The lower proportion of wood fiber in the WF/PS 25/75 mixture was equivalent to the density of PS. Fertilization with N did not affect the density of the growing media. The dry biomass of the cucumber plants correlated very strongly with the density of the growing media as the proportion of wood fiber in the growing media increased (Figure 3).

In their studies on growing media with wood fiber [40], the authors found that strawberries grown in a mixture of 75% wood fiber and 25% peat gave the best yields. This suggests that a mixture of growing media with wood fiber can improve plants’ performance. The composition of the growing media did not affect the chemical composition of the berries, but the berries of the plants grown in this mixture had a lower firmness than those grown in coir fiber. The most productive plants were in mixtures containing up to 75% wood fiber, even without a starter fertilizer [40]. Cucumbers were the most productive in a medium made of wood fiber 50% and peat 50% [29]. Other researchers’ studies on ornamental plants have shown that in growing media with 10% and 20% wood fiber, the leaf greenness index, the flower biomass, and the visual score did not differ from those grown on 100% peat substrate. A total of 40% wood fiber harmed all the growth parameters and the macro- and micronutrient contents of the leaves. The plants grown on a peat substrate enriched with 20% wood fiber and fertilized with nitrogen had the highest leaf greenness index, the highest number of flowers, and the highest N, P, Ca, Na, Fe, Mn, and Cu contents. This study showed that high-quality ornamental plants can be successfully grown in a growing media with 20% wood fiber and additional nitrogen fertilization [9]. In our experiment, the most productive cucumbers were grown in a WF/PS 50/50 mixture with additional N_13_ fertilizer. Higher nitrogen rates resulted in a lower fruit number and biomass per plant (Table 2).

Electrical conductivity (EC) is an indicator of the concentrations of soluble salts (Na^+^, Mg^2+^, Ca^2+^, Cl^−^, SO_4_^2−^, HCO^3−^, K^+^, NO^3−^, etc.) [41]. High EC has negative effects on germination, photosynthesis, plant vigor, yield, and the nutritional and economic values [42]. Salinity is detrimental to plant growth and development due to water stress, cytotoxicity due to excessive levels of ions such as sodium (Na^+^) and chloride (Cl^−^), and nutritional imbalances. In our study, it was found that at the highest N_30_ fertilizer rate, the dry biomass content of the aerial part of the plant was dependent on electrical conductivity (Figure 3). On the other hand, a very low EC value of the media indicates nutrient deficiency. During the anion exchange process, excess sodium ions can replace calcium and magnesium, leading to changes in the structure and fertility of the media. This results in the depletion of available nutrients. That different plants differ in their salt sensitivity-tolerance range [43]. Cucumber is a salt-sensitive species, and the EC of the growing media must be <2.7 dS/m, otherwise significant yield losses are inevitable. Our results also showed that EC was influenced by the biomass of fruit grown without additional nitrogen fertilization and at the lowest N_13_ rate (Figure 3). Zucchini (moderately salt-sensitive) grow well in a media with an EC of 2.6–2.8 dS/m, while tomatoes can tolerate salt concentrations of up to 2.9 dS/m with no yield loss [43]. An EC < 2.5 dS/m is considered optimal for creating an ideal substrate [44], but it has been reported that an EC < 3.5 dS/m is the most suitable for growing media and substrates with high EC have higher nutrient sorption and buffering capacities [39]. This study points out that the cucumber fruit biomass with increasing WF in the growing media was strongly dependent on EC, but only without additional fertilizer (NCF) and with the lowest N_13_ rate (Figure 3). When the nitrogen rate was increased to N_30_, a greater influence of EC was observed on the aboveground dry biomass than on the fruit biomass. The replacement of growing media with wood fiber for *Zinnia hibrida* and *Tagetes erecta* has been investigated [45]. The authors suggest that in media with wood fiber, fertilization with 100–200 mg L^−1^ N can maintain an adequate pH and EC levels of the substrate solution and plant growth without the need for additional N fertilization at the later growth stages.

In our experiment, increasing the proportion of wood fiber in the growing media increased the pH from 5.2 to 6.6, while additional N fertilization reduced the pH value by 7.8%. It has been pointed out that wood waste materials also have a naturally higher pH compared to that of peat, which requires less limestone for initial pH adjustment, but the effect on the substrate’s ability to protect itself against pH changes is unknown [46]. Another study also shows that the pH of the substrate solution decreases with an increasing N concentration [45]. Principle component analysis showed that the pH had no significant effect on the cucumber biometric parameters by increasing the proportion of wood fiber in the growth media (Figure 3). A moderately strong relationship was found between the fruit biomass on the plant and the pH when the plants were fertilized at the maximum rate of N_30_.

Inducing moderate salinity and/or nutritional stress and the biofortification of vegetables with micronutrients beneficial to human health (iodine, iron, molybdenum, selenium, silicon, and zinc) are well-known methods that have been successfully used to enhance the content of health-promoting phytochemicals in vegetables [47]. It has been shown experimentally that EC and the pH vary with the growing media, depending on the wood components, and can modify the chemical properties of the substrate differently. Other work has shown that increasing the ratios of some wood components increases the pH of the substrate and inhibits EC during the growing season [12]. Some researchers provided this conclusion after three seasons of tomato cultivation in three different substrates—wood fiber, coir, and perlite. The growing media did not show significant differences in tomato yields, but the yields were obtained slightly earlier in wood fiber [13,48].

Plants grown under controlled climatic conditions take up more nutrients from the growing media than those grown outdoors. The high nutrient requirement is due to the shallow penetration of the root system (limited by the volume of the vegetative receptacle), the weak suction power of the roots, and the large difference between the biomass of the aboveground part of the plant and the root system. The nitrogen concentration in wood fiber is generally low, so additional nitrogen is added either at the production stage or by the consumer. Therefore, the nutrient levels, especially nitrogen, can vary considerably depending on the production process. The addition of nitrogen during production can increase the level of water-soluble N. The levels of K, Na, Mg, and Ca can also vary considerably. Some wood fibers have been found to have slightly elevated P levels. The level of extractable (plant-available) nutrients is high, indicating that decomposition processes and nutrient exchange take place in wood fiber [49]. Wood fiber products used in combination with peat, the main component of the growing media, have been found to have a high water-holding capacity, which may have an impact on the retention of nutrients in the growing media [50]. Another aspect to consider is the efficiency with which these mineral nutrients can be used to achieve good production without using too much fertilizer, which can cause environmental pollution. In our experiment, at the end of the cucumber growing season, the levels of Cu, Mn, Zn, Fe, and B were found to be significantly higher in the WF/PS 50/50 mixture than those in the PS, the WF, and the WF/PS 25/75 mixture (Table 4). Only Cl was detected at higher levels in the WF growing media than those in the other media. Other researchers suggest that the accumulation of chloride should be attributed to unfavorable nutrients in the root environment [51]. Additional fertilization with N_30_ increased the levels of Cu, Zn, Fe, B, and Cl in the WF growth media. This may be related to the increase in EC, as the increase in N rate blocked the uptake of nutrients from the media, resulting in the highest micronutrient levels in the root zone. Sometimes, the Mn and Zn levels can vary over a wide range, but generally, the concentrations and availability of the other trace elements are very low. In general, the levels of trace elements in the WF media are higher than those of the water-soluble trace elements [11]. Scientists have investigated the chemical composition of nutrient solutions in the root environment of tomatoes grown in wood fiber and rockwool under different levels of nitrate nitrogen in nutrient solutions at 200, 220, and 240 mg N–NO_3_·dm^−3^. Despite the wide range of carbon-to-nitrogen ratios (C:N) in wood fiber, no significant variation in nitrate content in the root environment was observed. This was the result of an appropriate frequency of nutrient solution application per day [51]. Our results show that the N_min_ and the N_total_ increased significantly in the growing media (cucumber root zone), while C:N decreased significantly when the N fertilization rate was increased to N_23_ (Table 4). Although the N_min_ content in the WF media was highest at the end of the cucumber growing season, the C:N content was not as high as that in the WF media. The addition of wood fiber to the growing media may improve macronutrient uptake in the short term, but may reduce micronutrient uptake in the medium term, highlighting the complex effects of wood fiber on nutrient availability and plant growth [52]. The decreasing effect of N-NH_4_ and N-NO_3_ contents in wood fiber on cucumber growth can be attributed to biosorption due to the high C:N = 125 ratio in these media [53].

In our experiment, similar results were obtained; the highest C:N ratio was found in the wood fiber growing media compared to those of the peat substrate and the media mixtures, but additional N fertilization reduced this ratio. The N_total_ content was low in the WF media at the end of the growing season. The WF media had the highest N_min_, but there was no significant difference compared to that of the WF/PS 50/50 growing media. It can be concluded that for the cucumbers grown on WF, WF/PS 50/50 nitrogen uptake was more difficult than it was for the cucumbers grown on the PS and WF/PS 25/75 growing media. When fertilization was increased to N_23_ and N_30_, mineral N remained in the growing media (Table 4). Wright et al. (2008) found that plants grown on soils containing woody material require about 100 mg L^−1^ N more fertilizer to achieve a similar result to that of the plants grown on peat substrates. Different types of wood component also differ in their physical properties and their ability to immobilize N. The differences in air and water porosity, total water-holding capacity, and bulk density can also affect microbial activity and plant growth. The main cause of N immobilization is biosorption by microorganisms that use the N available in plants [54]. This can lead to nutritional deficiencies in ornamental plants and a reduction in their aesthetic value [50]. The negative effects of the sorption of N and other components on plant growth and flowering can be eliminated by appropriate fertilization. To overcome N immobilization in growing media with wood fiber, it is necessary to provide additional nutrient solutions from the beginning of plant growth [11,39,55].

Wood fiber-based growing media can be produced with the desired physical and chemical properties for the optimal growth of a wide range of plants and vegetables. Other studies on the yield of plants grown on wood fiber media show that additional fertilizers are needed, but growth may depend on the nature of the wood fiber and its content in the growing media. Wood fiber can be a reliable, consistent, renewable, and cost-effective alternative to the traditional peat substrate in greenhouses. The use of wood fiber in growing media is in line with sustainable development policies and can help reduce the carbon footprint of the product.

## 5. Conclusions

The cucumber fruit biomass and quantity were highest when the cucumbers were grown on the WF/PS 50/50 media. Additional fertilization with N_13_ significantly increased the cucumber yield. Higher nitrogen rates of N_23_ and N_30_ reduced the fruit biomass and the number of cucumbers per plant. Wood fiber reduced the density and increased the moisture content of the growing media. Increasing the nitrogen content decreased the moisture content of the growing media. Wood fiber reduced the acidity of the media. At the end of this study, significantly higher levels of Cu, Mn, Zn, Fe, and B were found in WF/PS 50/50 than those in the PS, WF, and WF/PS 25/75 media. The application of N_30_ fertilizer increased the levels of the trace elements Cu, Zn, Fe, B, and Cl in the EC growing media. It can be assumed that the excessive nitrogen applied blocked the uptake of nutrients from the media. At the end of the growing season, the micronutrient content in the root zone of cucumbers fertilized with N_23_ and N_30_ was highest. At the end of the growing season, the N_min_ content was higher in the growing media with a higher proportion of wood fiber, but the C:N content remained highest in the WF medium. The optimum medium with wood fiber for cucumber production in a greenhouse was found to be WF/PS 50/50 with additional fertilization with N_13_. Thus, wood fiber-based substrates can be produced with the desired physical and chemical properties for the optimal growth of a wide range of plants and vegetables.

Further research is needed to evaluate wood fiber-based soilless substrates containing different types of wood to assess not only N immobilization, but also the availability of other nutrients and the effect on the plants. A comparison of the range of wood production residues available from using different methods for the development of growing media is needed to show the advantages and disadvantages of wood products as substrate components. Cultivation technologies need to be developed based on nutrient management strategies that depend on the type of wood and the quantity used in the growing media. The cultivation technologies should be tested before making science-based recommendations to growers.

## Figures and Tables

**Table 1:** Characteristics of wood fiber and *Sphagnum* peat used in experiment as growing media and their parts.

Parameter	Wood Fiber	*Sphagnum* Peat
pH	4.6	5.3
EC mS m^−1^	58	76
C:N	435	80
N total % DW *	0.22	1.08
P mg kg^−1^ DW	57	181
K mg kg^−1^ DW	472	674
Mg mg kg^−1^ DW	29.3	23.2
Fe mg kg^−1^ DW	113.0	86.2
Cu mg kg^−1^ DW	2.9	3.0
Zn mg kg^−1^ DW	10.4	12.4
B mg kg^−1^ DW	15.3	14.0
Cl mg kg^−1^ DW	117.0	29.9

*—DW, these parameters were determined as dry weight.

**Table 2:** The indicators of cucumber productivity and the physicochemical properties of the growing media as a function of the nitrogen fertilization rate and the wood fiber content in the growing media.

Factors		Physicochemical Properties of Growing Media				Indicators of Cucumber Productivity			
Growing Media (A)	N Fertilization Rate (B)	Moisture, %	Density, g cm^−2^	EC, mS m^−1^	pH	Dry Biomass Content of the Plants, %	Aboveground Fresh Biomass, g per Plant	Number of Fruits, per Plant	Fruit Biomass, kg per Plant
Growing media									
PS		41.48 c	0.39 a	399 c	5.18 c	18.95 a	195.6 a	35.15 b	5.61 b
WF/PS 25/75		45.55 c	0.39 a	513 a	5.85 b	17.43 b	164.1 d	34.85 b	5.48 b
WF/PS 50/50		50.98 b	0.30 b	370 d	6.55 a	16.48 c	187.6 b	41.18 a	6.22 a
WF		55.99 a	0.23 b	420 b	5.98 b	14.30 d	172.0 c	36.80 b	5.68 b
Fertilization									
	CF	55.40 a	0.33 a	172 d	6.18 a	17.13 ab	212.9 a	36.61 b	6.06 a
	N_13_	54.90 a	0.33 a	434 c	5.88 b	17.25 a	204.0 a	40.00 a	6.03 a
	N_23_	49.14 b	0.33 a	522 b	5.80 b	16.13 c	157.5 b	34.55 b	5.40 b
	N_30_	34.56 c	0.33 a	575 a	5.70 b	16.65 b	144.9 c	36.82 b	5.51 b
Influence and interactions									
A		**	*	**	**	**	**	**	**
B		**	ns	**	**	**	**	**	**
A × B		ns	ns	**	ns	**	**	**	**

Note: peat substrate (PS); wood fiber/peat by volume (WF/PS 50/50); wood fiber/peat by volume (WF/PS 25/75); wood fiber (WF); conventional fertilization (CF). Data with the same letters for Factor A and Factor B are not significantly different at *p* < 0.05. * and **—the level of statistical significance at *p* < 0.05 and *p* < 0.01, respectively; ns—not significant.

**Table 3:** The variation (%) in micronutrient content in the growing media during the growing season, taking into consideration the amount obtained with fertilizer.

Growing Media	Fertilization Rate	Cu	Zn	Mn	Fe	B	Cl
PS	CF	−2	−67	−18	−65	1	853
	N_13_	−4	−66	−37	−80	21	586
	N_23_	16	−42	−36	−78	76	870
	N_30_	−11	−28	−26	−64	44	1044
WF/PS 25/75	CF	13	−45	−1	−59	31	14
	N_13_	7	−33	−9	−74	50	97
	N_23_	−3	−36	−2	−77	56	91
	N_30_	35	−24	−7	−59	102	120
WF/PS 50/50	CF	−31	−47	−14	−47	1	−9
	N_13_	−25	−52	−22	−49	26	14
	N_23_	−33	−48	−22	−63	36	119
	N_30_	−20	−40	−21	−63	81	144
WF	CF	−39	−62	−60	−86	−18	118
	N_13_	−39	−70	−77	−82	27	150
	N_23_	−39	−53	−71	−62	25	232
	N_30_	−39	−75	−78	−71	12	269

Note: a negative value indicates that the micronutrients were taken up by the plant. PS—peat substrate; WF/PS 50/50—wood fiber/peat by volume; WF/PS 25/75—wood fiber/peat by volume; WF—wood fiber; CF—conventional fertilization.

**Table 4:** The influence of the N fertilization rate and the wood fiber content of the growing media on the micronutrient content, the nitrogen content, and C:N at the end of the growing season.

Factor		Micronutrient Content of the Growing Media mg kg^−1^						N_total_ %	N_min_ mg kg^−1^	C:N
Growing Media (A)	N Fertilization Rate (B)	Cu	Mn	Zn	Fe	B	Cl			
Growing media										
PS		2.73 b	17.11 c	6.59 ab	27.25 c	18.98 b	281 b	1.28 b	136.4 bc	52.50 b
WF/PS 25/75		2.92 b	22.28 b	5.32 b	45.50 b	20.93 ab	312 ab	1.35 a	116.6 c	48.25 c
WF/PS 50/50		10.61 a	36.39 a	7.44 a	76.00 a	23.95 a	292 b	1.26 b	154.1 ab	47.92 c
WF		2.52 b	11.70 d	6.78 ab	34.58 c	17.08 b	342 a	0.88 c	186.6 a	94.75 a
Fertilization										
	CF	4.37 b	24.80 a	6.51 b	50.25 a	15.43 b	225 d	0.90 c	74.9 c	79.17 a
	N_13_	4.65 ab	20.38 b	5.70 c	41.75 b	19.58 a	262 c	1.05 b	155.4 b	62.75 b
	N_23_	4.25 b	21.63 b	7.14 a	42.33 b	22.05 a	344 b	1.37 a	224.5 a	51.75 c
	N_30_	5.52 a	20.68 b	6.78 ab	49.00 a	23.88 a	395 a	1.45 a	239.0 a	49.75 d
Influence and interactions										
A		**	**	*	**	*	*	**	**	**
B		*	*	*	*	*	**	*	**	**
A × B		*	*	*	*	ns	*	*	**	**

Note: peat substrate (PS); wood fiber/peat by volume (WF/PS 50/50); wood fiber/peat by volume (WF/PS 25/75); wood fiber (WF); conventional fertilization (CF). Data with the same letters for Factor A and Factor B are not significantly different at *p* < 0.05. * and **—the level of statistical significance at *p* < 0.05 and *p* < 0.01, respectively; ns—not significant.

## Data Availability

The data are contained within the article.
